# Health Convergence Between East and West Germany as Reflected in Long-Term Cause-Specific Mortality Trends: To What Extent was it Due to Reunification?

**DOI:** 10.1007/s10680-017-9455-z

**Published:** 2017-12-04

**Authors:** Pavel Grigoriev, Markéta Pechholdová

**Affiliations:** 1 Max Planck Institute for Demographic Research, Rostock, Germany; 2 University of Economics, Prague, Czech Republic; 3 Institut national d’études démographiques, Paris, France

**Keywords:** Mortality, East and West Germany, Convergence, Reunification, Causes of death

## Abstract

The sizeable mortality gap between the German Democratic Republic (East Germany) and the pre-unified Federal Republic of Germany (West Germany) narrowed rapidly after the two states were unified. Despite extensive research, the mechanisms underlying the convergence process are still not fully understood. Significant changes to coding practices and the system of data collection introduced in East Germany shortly after reunification have further complicated the ability of researchers to interpret mortality trends. Our aim is to assess the role of German reunification in the convergence process in light of the evolution of long-term mortality trends by causes of death. Compared to previous studies, we rely on much more detailed mortality data, which we first adjust for notable distortions. We propose an upward correction of cancer mortality, as well as corrections that account for obvious changes in the items selected within the ICD chapter of circulatory diseases. We identify three distinct processes that took place in East Germany around the time of reunification: (1) a sustained reduction in mortality that started before reunification; (2) a temporary increase in mortality in 1990–1991 that was related to the abrupt social transition, as reflected by socially sensitive causes such as accidents, alcohol-related diseases, and acute myocardial infarction; and (3) a reunification-driven process of convergence that was mostly caused by the accelerated decline in mortality from cerebrovascular and chronic heart diseases. Mortality improvements observed in the GDR starting in the 1980s might be interpreted as the first signs of a cardiovascular revolution. Shifts in individual behaviour likely started before reunification, whereas the real progress in medical care occurred later with the implementation of the Western system of health care. We therefore conclude that German reunification per se did not initiate the convergence process, but rather reinforced and accelerated trends that were already apparent.

**Electronic supplementary material:**

The online version of this article (10.1007/s10680-017-9455-z) contains supplementary material, which is available to authorized users.

## Introduction and Background

**Fig. 1:**
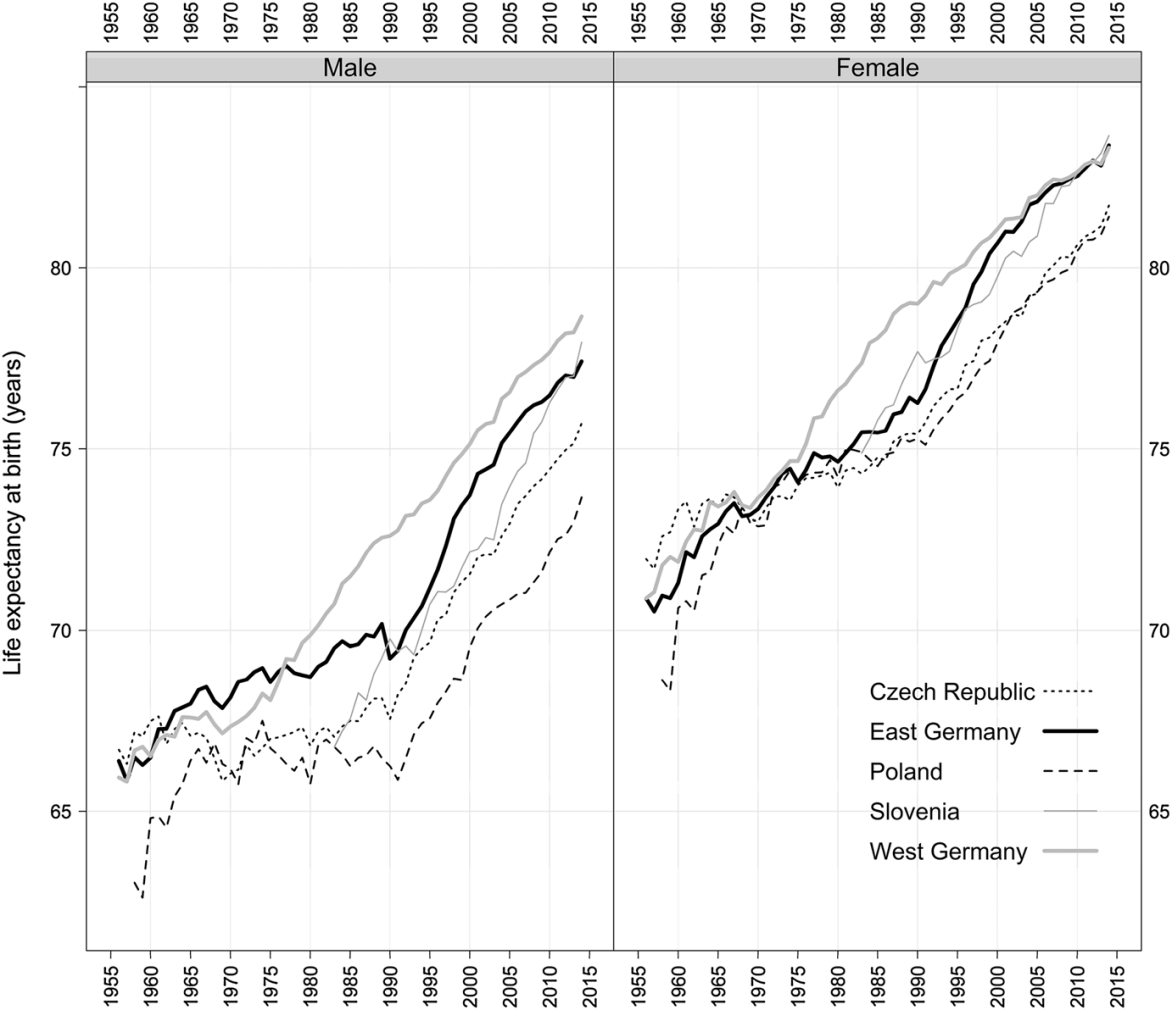
Life expectancy at birth by sex; East Germany, West Germany, and selected post-communist countries, 1956–2014. *Source*: Human Mortality Database

Between 1949 and 1989, Germany was divided into two separate states: the socialist GDR (German Democratic Republic) and the free market-oriented FRG (Federal Republic of Germany); hereafter, East and West Germany. The sizeable mortality gap between the two German states that accumulated during the period of the separation narrowed rapidly after reunification (Fig. 1). The post-unification convergence has been particularly pronounced among women: today, female life expectancy is even slightly higher in East than in West Germany. Among males, the East–West gap had narrowed by the late 1990s, but the mortality differences between the two German parts have persisted, and male life expectancy shows no signs of converging in the near future (Fig. 1).

German reunification is often described in the literature as a ‘natural experiment’ that provides a unique opportunity to disentangle the effects of political and financial interventions on population health (Nolte et al. 2000b; Gjonça et al. 2000). After four decades of living under a sharply different political, social, and economic system, East Germans had to adapt very quickly to the Western socio-economic system when the two parts of Germany reunited in 1990. This reunification process was accompanied by the massive Western investments in the infrastructure of East Germany, including the social security and health care systems (Vogt 2013; Vogt and Kluge 2015). Studies that focused on the mortality effects of German reunification have shown that the rapid progress in health observed in East Germany was mainly driven by the reduction in mortality from cardiovascular diseases (Nolte et al. 2000b; Luy 2004). The adoption of the FRG health care system, which gave East Germans improved access to modern drugs and technologies, is reasonably seen as the most obvious explanation for this progress (Nolte and McKee 2000; Vogt and Vaupel 2015).

Among other reunification-related factors, the improvement in living standards, especially through social welfare and pension benefits, has been cited as an important contributor to the reduction in mortality, even at very advanced ages (Vaupel et al. 2003). Behavioural factors—such as dietary habits, smoking, and alcohol—have received less attention (Gjonça et al. 2000; Nolte and McKee 2000, Myrskylä and Scholz 2013). Some scholars have, however, suggested that behavioural factors are important determinants of the East–West mortality divide (Bobak and Marmot 1996) and that these factors have been largely responsible for the health progress that has been observed in some other post-communist countries (Zatoñski and Boyle 1996; Bobak et al. 1997).

Psychosocial stress has also played a role in the reunification process. Based on the assumption that populations living under totalitarian regimes or facing dramatic socio-economic changes and uncertainty have elevated mortality risks (Bobak and Marmot 1996), a number of scholars have argued that some portion of the mortality decline in East Germany in the 1990s might be explained by the reduction in the psychosocial stress associated with the less favourable socio-economic and political conditions in the GDR (Diehl 2008). By contrast, other scholars have suggested that stress resulting from the fall of the Berlin Wall in 1989 contributed to the short-term increase in adult male mortality in 1990 (Häussler et al. 1995). Additionally, some researchers have pointed out that mortality among young East German males rose in part because of an increase in transport accidents, as many East Germans gained access to motor vehicles for the first time and were driving on poorly maintained roads (Winston et al. 1999; Clark and Wildner 2000). A number of studies have cited the ‘statistical artefact explanation’ as a possible contributor to the rapid decline in mortality in the former GDR (Eberstadt 1994; Häussler et al. 1995; Diehl 2008). It is very likely that the massive migration flows to the West around 1990 were underestimated in the official statistics, which resulted in the overestimation of the population denominator and, consequently, the underestimation of the true mortality rates. Nevertheless, the overall impact of this factor on mortality is generally considered to be small or negligible (Nolte et al. 2000a).

Most explanations for the growing mortality gap between East and West Germany before reunification, and its steady contraction thereafter, have revolved around differences and changes in two major factors: health care and lifestyles (Heinemann et al. 1996; Nolte et al. 2000b; Nolte and McKee 2000, Nolte et al. 2002; Rossnagel et al. 2003). Psychosocial factors and living standards have often been discussed as supplementary explanations (Kibele 2012). As it is clear that a complex interplay of these factors was responsible for the rapid convergence process, it is hardly feasible to quantify the relative contributions of single factors. The precise mechanisms underlying the marked improvement in health in East Germany are still not fully understood. This is partly due to the lack of availability of detailed long-term mortality statistics that are of good quality and are internationally comparable.

In this paper, we assess the role of German reunification in the East–West health convergence through a comparative analysis of long-term mortality trends by causes of death. Compared to previous studies, we rely on much more detailed mortality data, which we first adjust for notable distortions. These newly produced data enable us to make a more accurate assessment of the factors that have contributed to the rapid convergence in the mortality levels of the two parts of Germany.

## Data

### New Datasets

The cause-specific mortality data were provided by the German statistical office in either paper or electronic format and were computerised, checked, and standardised at the Max Planck Institute for Demographic Research. Our analysis is based on the data reconstructed over the last two revisions of the International Classification of Diseases: the ICD-9 and the ICD-10. In 1979, the two countries adopted the ICD-9 simultaneously. However, in 1979 an abridged list containing just 35 items was being applied in the GDR, and only 533 items were being used in the FRG. Starting in 1980, three-digit ICD-9 classifications were applied in both countries. The ICD-10, which provided detail up to at least three digits, was adopted in 1998.

Table 1 provides an overview of the classifications of causes of death that were in use in the GDR (East Germany) and the FRG (West Germany) starting in 1952. Interestingly, before reunification, these revisions were implemented in the same years: 1952, 1968, and 1979.

**Table 1:** Classifications of causes of death used in the GDR and the FRG and the number of available items in parentheses

	1952–1968	1969–1978	1979	1980–1990	1991–1997	Since 1998
GDR/East Germany	GDR-52 (301)	ICD-8 East (147)	ICD-9 East (35)	ICD-9 East (full list)	ICD-9 (full list)	ICD-10 (full list)
FRG/West Germany	DAS-52 (447)	ICD-8 (448)	ICD-9 (533)	ICD-9 (full list)		

The cause-specific time series were previously reconstructed at the three-digit ICD-9 level for the period covered by the ICD-8 and the ICD-9 in West Germany (Pechholdová 2008, 2009). Later on, the FRG series were extended to the ICD-10 based on a specific shortlist of 186 items (Pechholdová 2011). Currently, the continuous time series are being reconstructed for both countries for the period starting in 1960.

Regarding the population counts, the census of 2011 was the first census that had been conducted in Germany since the censuses of 1981 in the GDR and of 1987 in the FRG. Because the risk of statistical error (mostly due to the incorrect registration of migration events) increases progressively with time, this unusually long inter-censal period resulted in a decrease in the quality of the population estimates. The results of the last census revealed that the population of Germany was actually around 1.5 million lower than the population estimates based on the 1980s censuses and on the data on births, deaths, and migration. This study relies on the revised data on population exposures produced for both East and West Germany within the framework of the Human Mortality Database Project (Kluesener et al. unpublished manuscript).

### German Specifics of Data Collection and Coding

There were substantial differences in approaches used to collect cause-of-death data in East and West Germany prior to reunification. The GDR followed a model that was prevalent in many Eastern European countries, in which authorised physicians were responsible for both issuing the death certificate and coding the underlying cause of death. Physicians were instructed to minimise coding to the ICD chapter of ill-defined diseases. The death certificates were collected at local registry offices, where they were checked and recorded. The death records (along with the original certificates) were then forwarded to the Federal Central Statistical Office (*SZS*—*Staatliche Zentralverwaltung für Statistik*). The GDR system of death registration was centralised and reliable (Scholz et al. 2016). However, during the period when the ICD-9 was in use, selected causes of death (most digestive diseases, ill-defined causes, and a large share of accidents) were considered politically sensitive and were thus excluded from the official statistical reports, while a separate set of ‘hidden’ forms that listed the real causes of death in these cases were kept in a secret archive. This practice may have been inspired by a similar practice adopted by authorities in the USSR in the early 1970s (Meslé et al. 1992). Fortunately, we were able to retrieve these ‘hidden causes’ from the archives and to add them to our dataset.

In the FRG, medical doctors diagnosed only pathologies and provided the chain of morbid events, expressed as the order of the diseases, on the death certificate. After the plausibility of the medical information provided was verified at the local public health department, each death record was transferred to the respective regional statistical or health office, where it was encoded by specially trained staff (Pechholdová 2008). The national statistics were then summarised from the regional data. This system of data collection, classification, and coding in West Germany (and in East Germany since reunification) has been described in greater detail elsewhere (Schelhase and Ruebenach 2006; Schelhase and Weber 2007; Pechholdová 2008).

### Territorial Coverage: The Berlin Issue

In addition to the data collection issues described above, we had to deal with inconsistent territorial coverage due to the prior division of Berlin into East and West. Until 1998, the territory designated as the GDR/East Germany consisted of five *Länder* (*Mecklenburg*-*Vorpommern, Brandenburg, Sachsen*-*Anhalt, Sachsen, Thüringen*) and East Berlin. In 1998, the official German statistics ceased tabulation of cause-of-death data for East and West Berlin separately and have since provided data for Berlin as a whole only. Using the available regional data on raw death counts, we have designed our dataset in the following way: for the 1960–1997 period, we keep the historical division (East Germany incl. East Berlin and West Germany incl. West Berlin), whereas for the period starting in 1998, we incorporate the data for West Berlin into the data for East Germany. As the mortality differences between East and West Germany—and, presumably, East and West Berlin—had diminished substantially by 1998, incorporating the West Berlin data into the data for East Germany should not hamper the interpretation of mortality trends. To achieve the desired territorial coverage, we combine the data on population exposures from the HMD with the data from the Human Fertility Database (HFD) since 1998. As the HFD provides data on Germany, East Germany (without East Berlin), and West Germany (without West Berlin), it allows us to derive data on population exposures for Berlin.

### Data Quality Issues

In the GDR, the WHO’s rules were not always strictly followed, as medical doctors were charged with both selecting and encoding the underlying cause of death (Brückner 1993). Several studies have noted that the cause-specific mortality structure reported in the GDR differed from the structure reported in West Germany (Brückner 1993; Häussler et al. 1995; Hoffmeister and Wiesner 1993; Modelmog et al. 1992). The degree of misreporting is particularly noticeable for circulatory diseases and cancer (Brückner 1993), with the first group of causes being over-reported, and the second group of causes being under-reported (Dinkel 1999; Luy 2004; Heinemann et al. 1998). Our investigation of the death certification practices in the GDR showed that physicians were required to complete an additional form when the patient died of cancer. Some share of the under-reporting of cancer in the GDR might thus be explained by the reaction of the certifying physicians to this requirement, i.e. physicians may have certified another cause simply to avoid this extra administrative burden. Another possible reason for the under-registration of cancer deaths in East Germany is related to the tendency in the GDR (and in other countries of Eastern Europe) to report the most immediate cause of death (very often a cardiovascular cause) rather than the underlying cause of death. Finally, the comparability of the GDR mortality data was affected by the use in East Germany of a more restrictive definition of live birth (the newborn was counted as a live birth if both a heartbeat and respiration were detected), which resulted in a systematic underestimation of infant mortality. While the WHO coding rules were followed more strictly in the FRG, because the production of cause-specific statistics was decentralised, regional coding practices may have affected the underlying cause-of-death selection (Bubenheim 2000).

## Methods

### Methods Related to Data Treatment

#### Dealing with the Coding Change of 1990

On 1 October 1990, a new coding system was introduced in East Germany that was intended to harmonise the cause-of-death registration in the two parts of Germany. The implementation of this system led to severe disruptions in the reporting of a significant number of the causes of death between 1990 and 1992. In addition, several real processes affected mortality trends in the beginning of the 1990s: massive investments in the health care system, the increase in exposure to traffic accidents, and the increase in social stress related to the socio-economic transformation. Due to the complexity and the historical coincidence of these processes, it is unrealistic to believe that we can fully separate the pure effects of reunification from the effects of the coding changes. Using data-driven assumptions, we are proposing a plausible approach to correcting for the coding-related cause-specific mortality changes between 1990 and 1992 (see the online supplementary materials).

#### The Shortlist of 186 Causes of Death

To resolve the data collection problems that arose in the periods following the transition to a new coding system and the transition from the ICD-9 to the ICD-10, we refer to an abridged list of 186 causes previously proposed by Pechholdová (2011). The list—the design of which was based on previous experiences with the reconstruction of time series in several other countries—has five specific purposes: (1) to allow for a smooth linking of the ICD-9 and the ICD-10, (2) to allow for maximum comparability between countries, (3) to make the list compatible with other shortlists currently in use, (4) to acknowledge the historical epidemiological context, and (5) to ensure that we could keep most of the explanatory information.

The primary purpose of the list was to facilitate a smooth transition from the ICD-9 to the ICD-10. The list is compatible with the ICD-9 basic tabulation list (BTL), the ‘European shortlist’ of 65 causes (by EUROSTAT), the NCHS list of 113 selected causes of death (Anderson et al. 2001), and a shortlist proposed by Vallin and Meslé (1988). The list was also designed to allow for the study of several medical concepts, such as the idea of avoidable causes of death, which was conceived to measure the performance of the health care system (Nolte and McKee 2004), and the concepts of smoking- (Colditz 2000) and alcohol-attributable mortality (Ridolfo and Stevenson 2001). See "Appendix 1" for the full list and the correspondences between the ICD-9 and the ICD-10.

#### Bridging ICD-9 and ICD-10

The ICD-10 was adopted in Germany in 1998. To bridge the ICD-9 and the ICD-10, we are using the correspondences defined along with the 186 items of the selected shortlist. As the change in the classification in Germany was not accompanied by other changes to the coding system, the effect of the ICD-10 on time comparability was minor, and the applied correspondence table yielded continuous time series without the need for any major corrections.

### Methods for Analysing Mortality Trends

The mortality rates were calculated on the basis of the reconstructed data and the revised data on the population exposures and were then standardised using the WHO European population standard (Waterhouse et al. 1976). Before that point, the deaths attributed to ill-defined conditions (which represented very small shares of all causes in both East and West Germany) were redistributed proportionally among other causes. To determine the contributions of the age groups (aggregated into six broad age groups: 0–14, 15–29, 30–44, 45–59, 60–74, and 75 +) and the causes of death responsible for differences in life expectancy at birth, we relied on Andreev’s life table decomposition technique (Andreev 1982). The required life tables were calculated on the basis of the reconstructed data.

The analysis of mortality trends before 1980 was based on the provisional reconstructed data and was thus performed at the level of large classes only. To obtain these series, we first accounted for the transition from the GDR-52 classification to the abridged ICD-8 using the Meslé–Vallin reconstruction method and only then aggregated the harmonised data into classes. The direct aggregation of the raw data for the period of the GDR-52 classification (for the 1960–1967 period) was not possible because of the specifics of the old GDR-52 classification. The comparison of these data with the corresponding West German data was possible only from 1968 onwards because of the limited availability of the reconstructed cause-specific mortality data for the FRG (Pechholdová 2009).

## Results

### General Mortality Trends

**Fig. 2:**
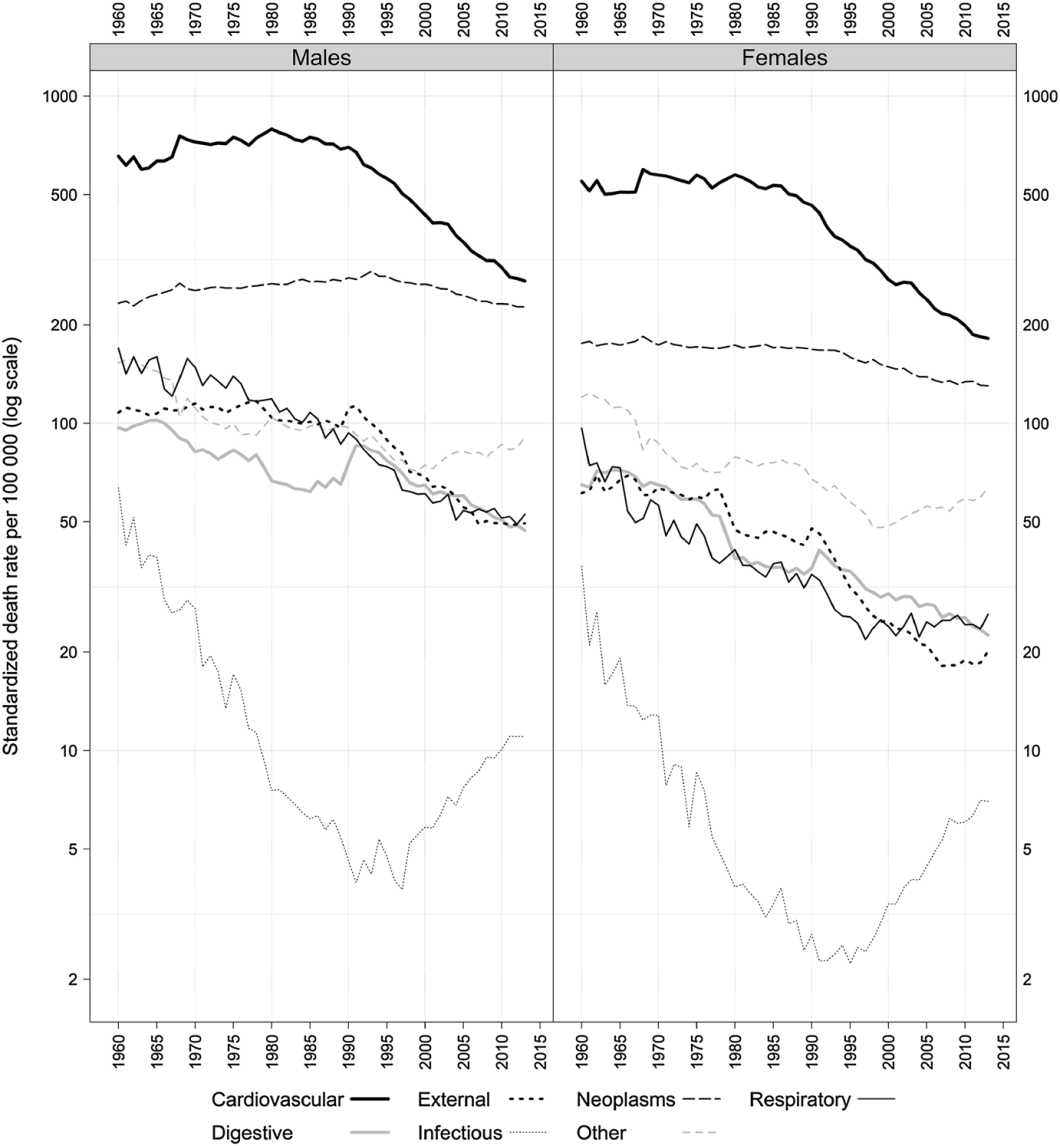
Mortality trends by main causes of death in East Germany, 1960–2013. *Source*: Reconstructed mortality series by causes of death. *Note*: the reconstructed data before 1980 are only preliminary

Figure 2 depicts the mortality trends in East Germany by broad classes of causes of death over the period 1960–2013, plotted on a log scale. It is not surprising to see that mortality from cardiovascular diseases (CVD) and neoplasms were the leading causes of death for both men and women. After a notable jump in 1968 that is attributable to the effects of the massive Hong Kong influenza pandemic (*Akademie für Ärztliche Fortbildung* 1972), CVD mortality stagnated up to 1980. After that point, it started decreasing slowly, with the decline accelerating in the 1990s. Cancer mortality also started falling at the beginning of the 1990s, though the decline in deaths from cancer has been much slower than the decrease in CVD mortality. As a result of these trends, the mortality levels for the two leading causes of death are now rather similar.

The trends in other causes of death prior to reunification have generally been positive. Between 1960 and 1990, there has been an impressive tenfold reduction in mortality from infectious diseases and a continuous decline in mortality from respiratory diseases. These trends reflect the epidemiological transition (the process of the replacement of infectious diseases by chronic diseases due to improved sanitation and treatment), which was reinforced by the relative effectiveness of the GDR’s socialist health care system in tackling communicable diseases.

Several negative developments after reunification should be noted. First, mortality from infectious diseases started increasing in both men and women. By 2013, mortality from this cause was more than twice as high as it was in 1990. Furthermore, mortality from digestive diseases and external causes increased markedly in the early 1990s. The increase in mortality from digestive diseases was particularly pronounced. However, it is worth noting that the mortality levels reported for this cause of death during the 1980s were suspiciously low relative to the levels reported in the 1960–1970s and the 1990s. The fact that the period of trend disruption coincides with the period of time when the ICD-9 (East) was in use in the GDR suggests that these mortality levels may have been underestimated.

**Fig. 3:**
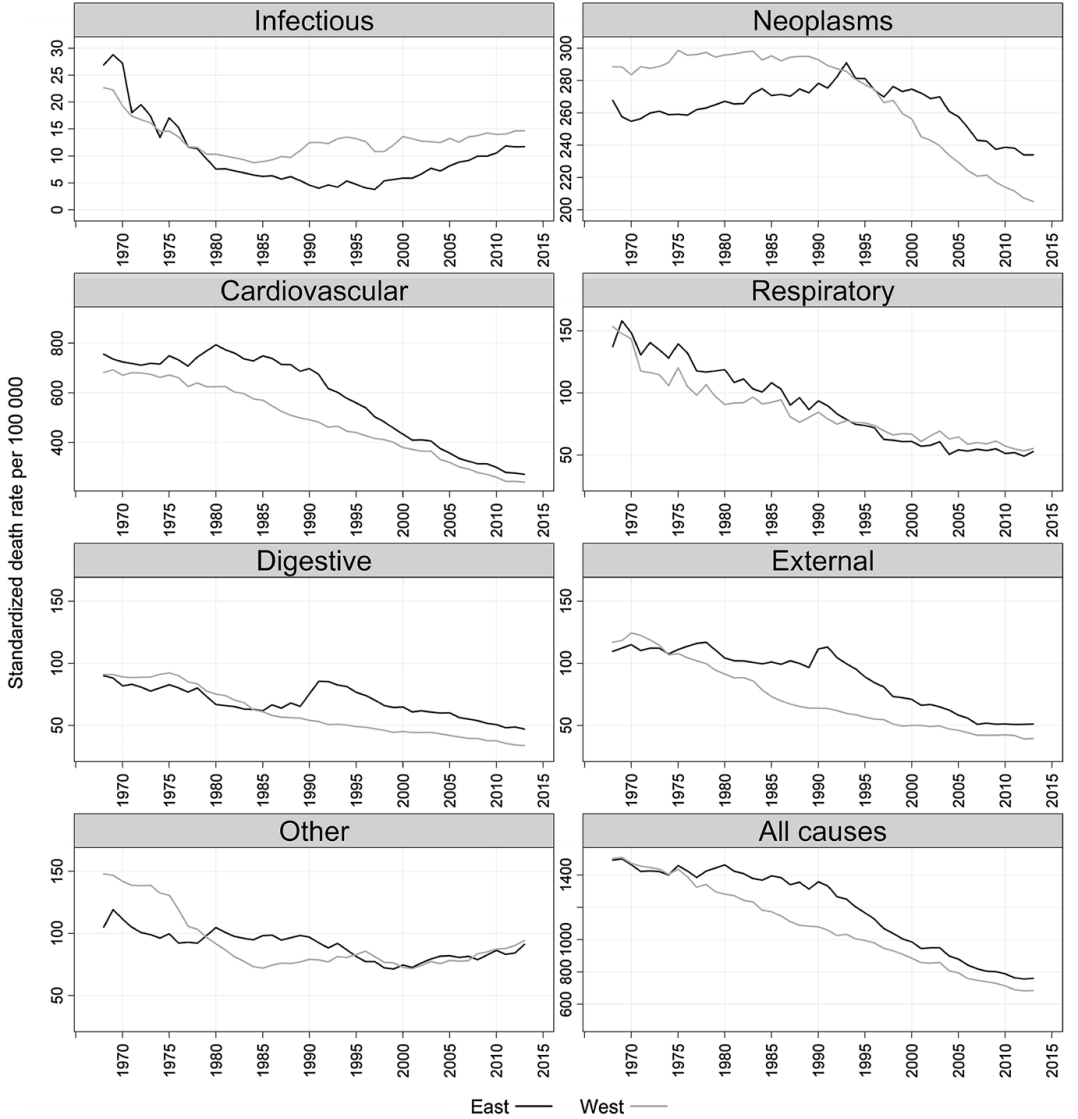
Mortality trends by main causes of death in East and West Germany, 1968–2013, males. *Source*: Reconstructed mortality series by causes of death. *Note*: the reconstructed data for East Germany before 1980 are only preliminary

**Fig. 4:**
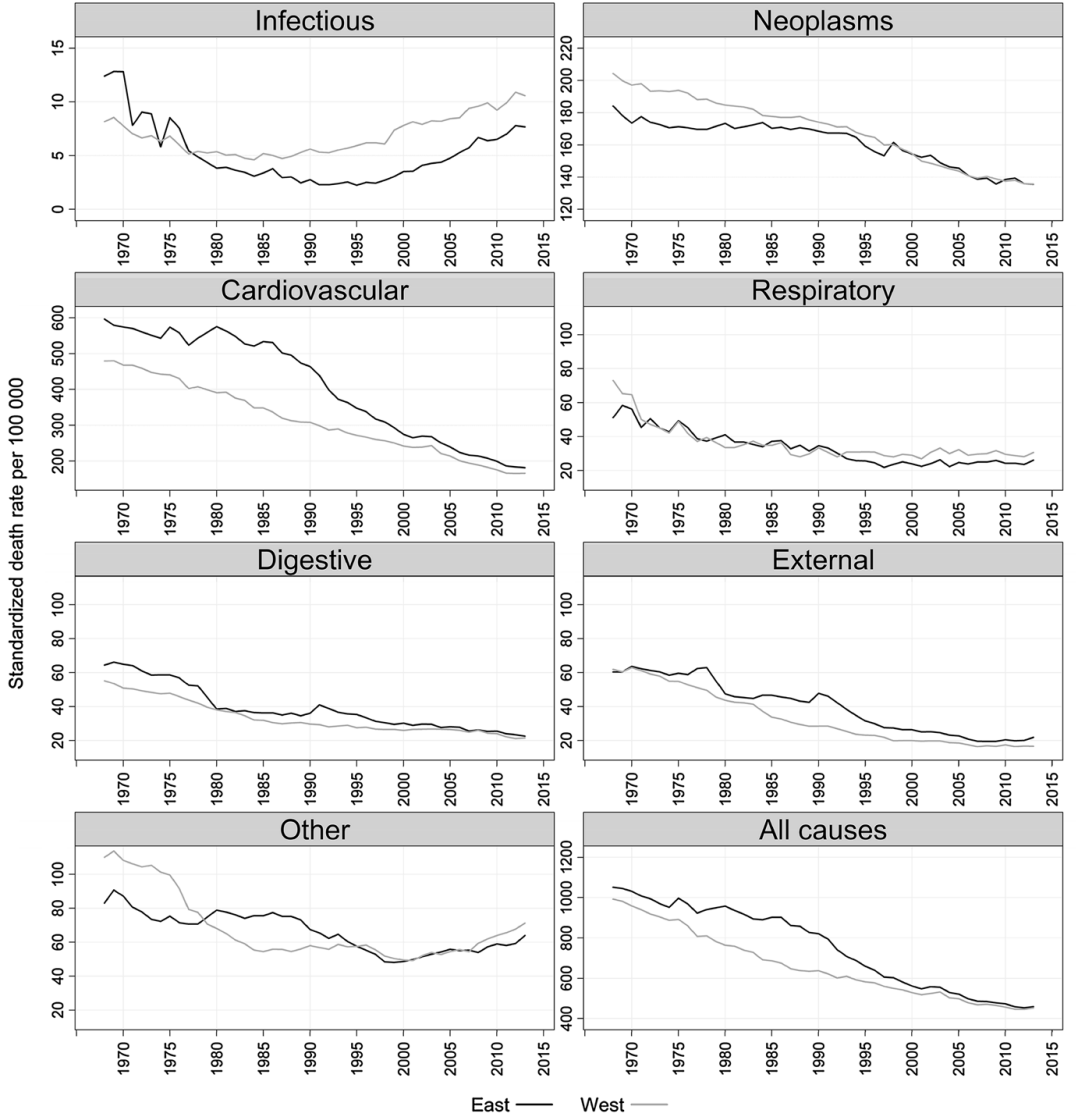
Mortality trends by main causes of death in East and West Germany, 1968–2013, females. *Source*: Reconstructed mortality series by causes of death. *Note*: the reconstructed data for East Germany before 1980 are only preliminary

Problems with the quality of cause-of-death registration in East Germany can be better understood if the East German trends are compared to the West German trends (Figs. 3, 4).

The data on the trends in mortality from digestive diseases are questionable. Unexpectedly, the data show that up to 1985, the observed mortality from this cause among males was higher in West than in East Germany. The data further indicate that the reverse trend occurred among women, but that this gap disappeared with the implementation of the ICD-9. The level of mortality from digestive diseases in East Germany increased markedly, first in 1990 and then in 1991 as the FRG coding practices were being adopted. The historical trends in digestive disease mortality were disrupted at the time of the implementation of the ICD-9 in 1979: when compared to the mortality levels reported in the ICD-8 period, the mortality levels reported in the ICD-9 period appear to be underestimated. The practice of hiding the real cause of death may be one factor in this underestimation, as a similar but less pronounced time pattern was observed for external causes of death (another hidden aetiological group). It is possible that the awareness of the undesirability of reporting these diseases as the underlying cause of death motivated the certifying practitioners to select another condition. This bias probably affected the reporting of circulatory diseases and some types of neoplasms (infectious, respiratory, and other diseases were unaffected by the revisions of the classification). Like for neoplasms, a thorough reassessment of the reporting of these groups of diseases is needed to ensure historical continuity.

The increase observed in 1990–1991 is partly attributable to the artefact of the under-reporting of digestive (and external) diseases before 1990 and the consequent coding change in 1990. However, our analysis of detailed trends by age and sex (not shown here) revealed that at least half of the increase observed in 1990–1991 was real.

One important point should be kept in mind when tracing the evolution of mortality from other causes of death and of overall mortality: namely that the East–West mortality gap, which started growing in the late 1970s, was driven by a continuous reduction in CVD mortality in West Germany that was not accompanied by a corresponding reduction in the East. This is especially notable given that a portion of the ICD-9-related increase in 1979–1980 in East Germany was an artefact of the previously mentioned coding practices. Thus, in the following analyses we focus on the period after 1980, during which a substantial share of the historical convergence of mortality trends in East and West Germany occurred.

### Evolution of the East–West Gap in Life Expectancy by Age and Cause of Death

The results of the decomposition analysis are presented in "Appendix 2". Here, we show the ages and the causes of death responsible for the differences in life expectancy at birth in East and West Germany. Positive values indicate a West German advantage, while negative values indicate an East German advantage.

In 1980, the East–West gap in male mortality of 1.17 years was determined mainly by men aged 60 and above, who contributed 0.83 years. The lower mortality from heart diseases in West Germany was mostly responsible for the disadvantage among Eastern males (0.59 years). Differences in rates of mortality from infectious and respiratory diseases contributed 0.24 years in favour of Western males. The situation changed dramatically by 1990, when the mortality gap reached a historic maximum of 3.39 years. At this point, mortality among people at young and adults ages became particularly important. The age groups 15–44 were responsible for 0.90 years of the disadvantage among Easterners, and the age groups 45–59 contributed one year to the gap. Unlike in 1980, mortality from external causes was an important contributor (0.94 years). The gap in 2013 (1.21 years) was even slightly larger than the gap in 1980. While mortality associated with alcohol consumption (external causes and digestive diseases) still played a significant role in the gap, the contributions of heart diseases and cancers became even more important (0.57 and 0.36 years, respectively).

The East–West mortality difference among females developed somewhat differently. In 1980, the disadvantage among East German females was twice as high as it was among males (2.10 vs. 1.17 years). Like among males, this gap among females was determined by mortality from cardiovascular diseases at older ages. By 1990, the disadvantage among Eastern females had increased to 2.77 years. This increase was, however, much more modest than the increase among males. It is worth noting that the contribution of mortality from external causes of 0.40 years was in favour of Western females. By 2000, the gap in female mortality between East and West Germany was just 0.5 years, and by 2013, the gap had almost disappeared (0.01 years).

**Fig. 5:**
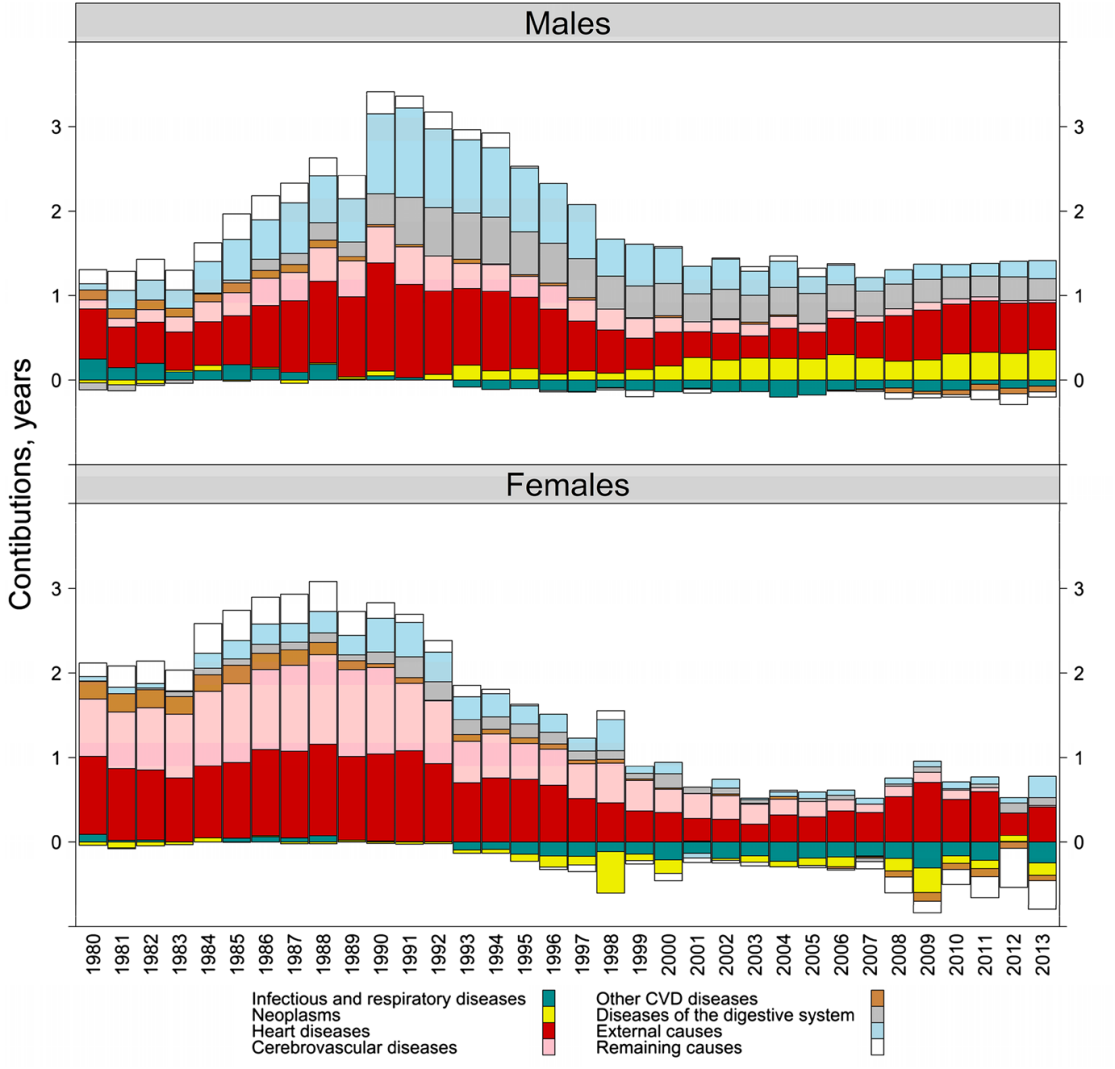
Cause-specific contributions to East–West gap in life expectancy at birth, 1980–2013. *Source*: Own calculations based on the reconstructed data

**Fig. 6:**
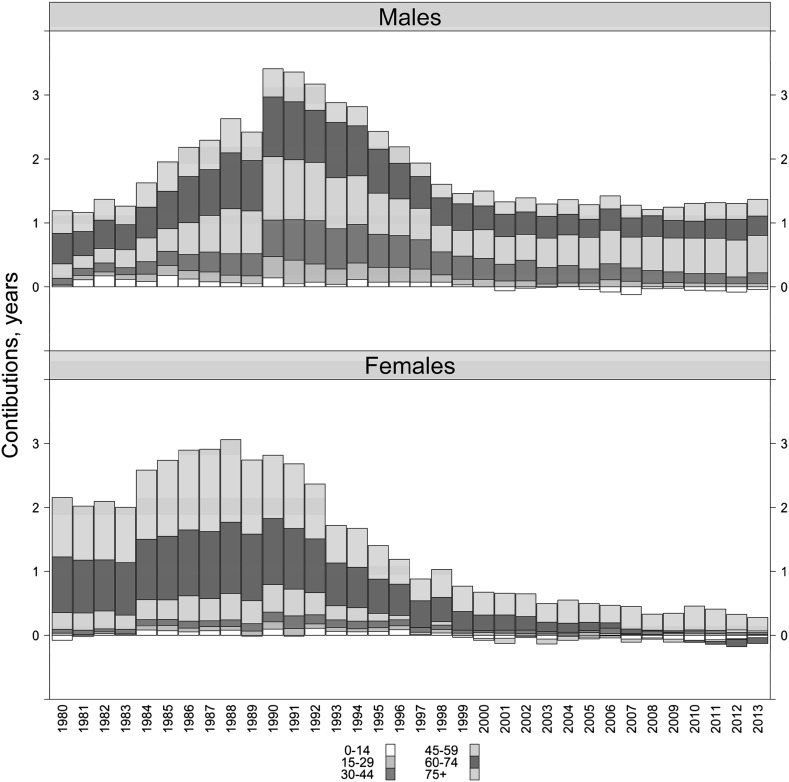
Age-specific contributions to East–West gap in life expectancy at birth, 1980–2013. *Source*: Own calculations based on the reconstructed data

Figures 5 and 6 depict the yearly evolution of the East–West mortality gap by causes and ages by each single year since 1980.

It should be noted that until the mid-1980s, the East–West mortality gap held steady at about the same level: i.e. around 1 year for males and 2 years for females. The gap started growing in the late 1980s, a period when political and social changes were occurring in the GDR. Historically, the biggest gap between East and West was observed in the years around reunification; not in the 1970s or the early 1980s.

During the years immediately after reunification, the role of mortality from external causes of death among adult males became particularly important. A decade later, when the gap had grown smaller and had stabilised, these causes of death lost their importance. Over the past decade, external causes have not been among the major contributors to the East–West mortality gap.

In more recent years, the persisting gap in male mortality has been driven by higher mortality from heart diseases and cancers in East Germany. The 45–59 age group is mostly responsible for the disadvantage among Eastern males. Among females, mortality from heart diseases at ages 75 and older has been the major contributor to the remaining small difference.

### Cause-Specific Mortality Trends

In this section, we analyse mortality trends from some specific causes of death. We assume that the mortality trends in selected causes reflect important changes in both individual behaviour and the quality of health care. Alcohol-related causes and accidents have been selected to reflect lifestyle changes related to the rapid transition to the new political and socio-economic system. In absolute terms, cardiovascular conditions have been the leading causes of death associated with both the progress in medical care and individual lifestyles. Finally, causes of death amenable to health care can provide a general assessment of the performance of the health care system.

#### Alcohol-Related Causes and Accidents

**Fig. 7:**
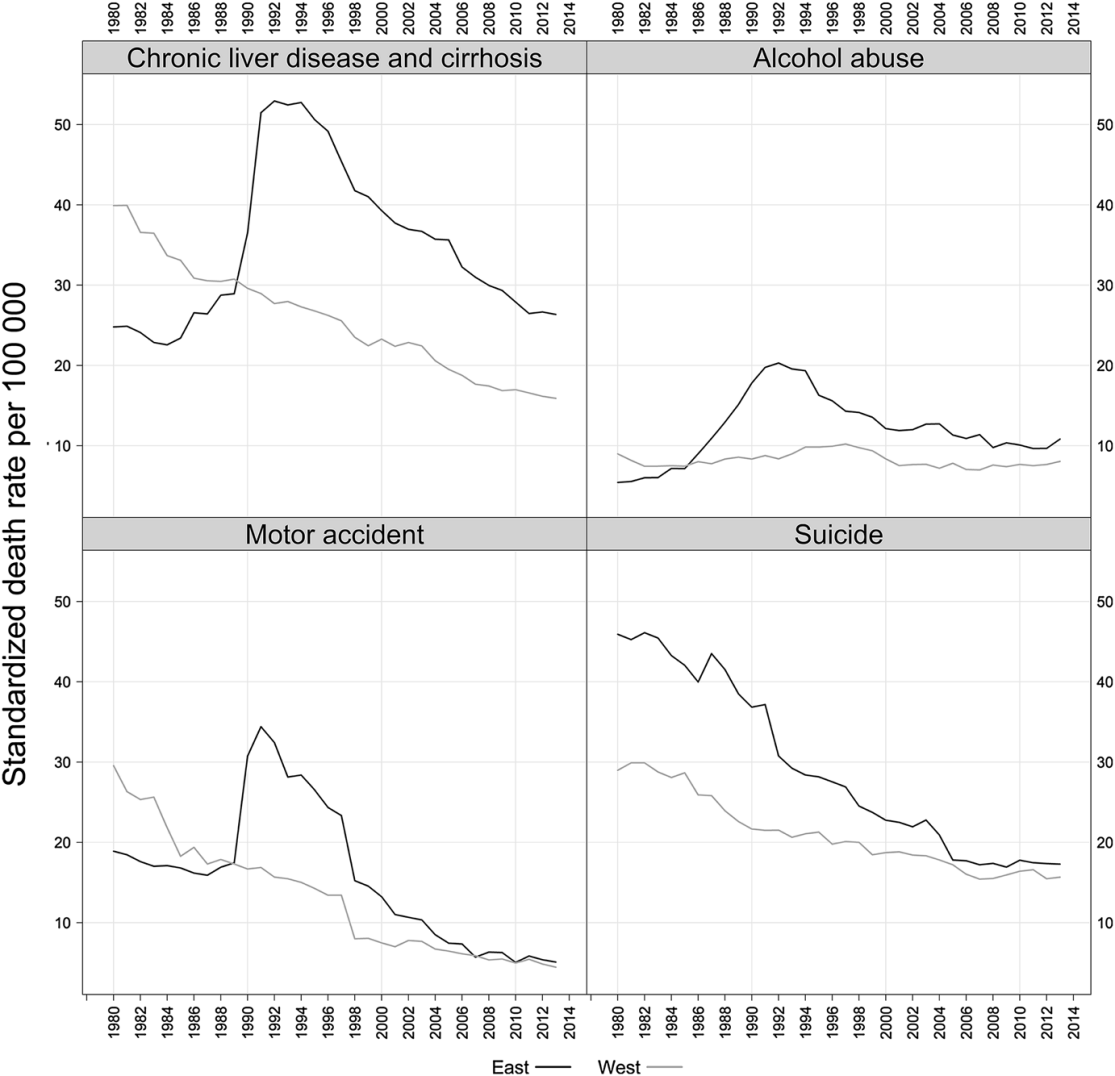
SDR from selected alcohol-related and external causes of death, East and West Germany, 1980–2013, males. *Source*: Reconstructed mortality series by causes of death

As was shown above, mortality from external causes of death and mortality from diseases of the digestive system were important contributors to the growing East–West mortality gap among males during the years immediately after reunification. Both causes of death are also known to be closely related to alcohol consumption (WHO 2014). Figure 7 displays the dynamics of male mortality from chronic liver diseases and cirrhosis (the main cause of death in the chapter of digestive diseases), alcohol abuse, motor transport accidents, and suicide.

Mortality from the causes of death that are directly attributable to alcohol consumption (liver cirrhosis and alcohol abuse) has increased rapidly since the late 1980s. The increase in mortality from liver cirrhosis has been particularly noticeable. A similar pattern of an abrupt increase in mortality can be seen when we look at mortality from transport accidents in the early 1990s. However, somewhat unexpectedly, suicide mortality did not change at all during the years of the socio-economic and political transition; generally, mortality from this cause of death declined rapidly until it converged to the level observed in West Germany in the mid-2000s. A similar pattern of convergence can be seen in mortality from transport accidents and alcohol abuse, but not in mortality from liver cirrhosis.

The results of the decomposition analysis (not shown here) confirm the importance of alcohol-related causes and accidents in the decrease in male life expectancy of 0.9 years in East Germany between 1989 and 1990. About 60% of this decline was determined by the increase in mortality from external causes (− 0.42 years) and mortality from diseases of the digestive system (− 0.15 years). Almost all of the remaining difference (− 0.27 years) was due to mortality from heart diseases, especially acute myocardial infarction, which can be partially attributable to alcohol consumption. Overall, the group of males under age 40 alone contributed roughly half of the total decrease in life expectancy at birth between 1989 and 1990.

#### Cardiovascular Diseases

**Fig. 8:**
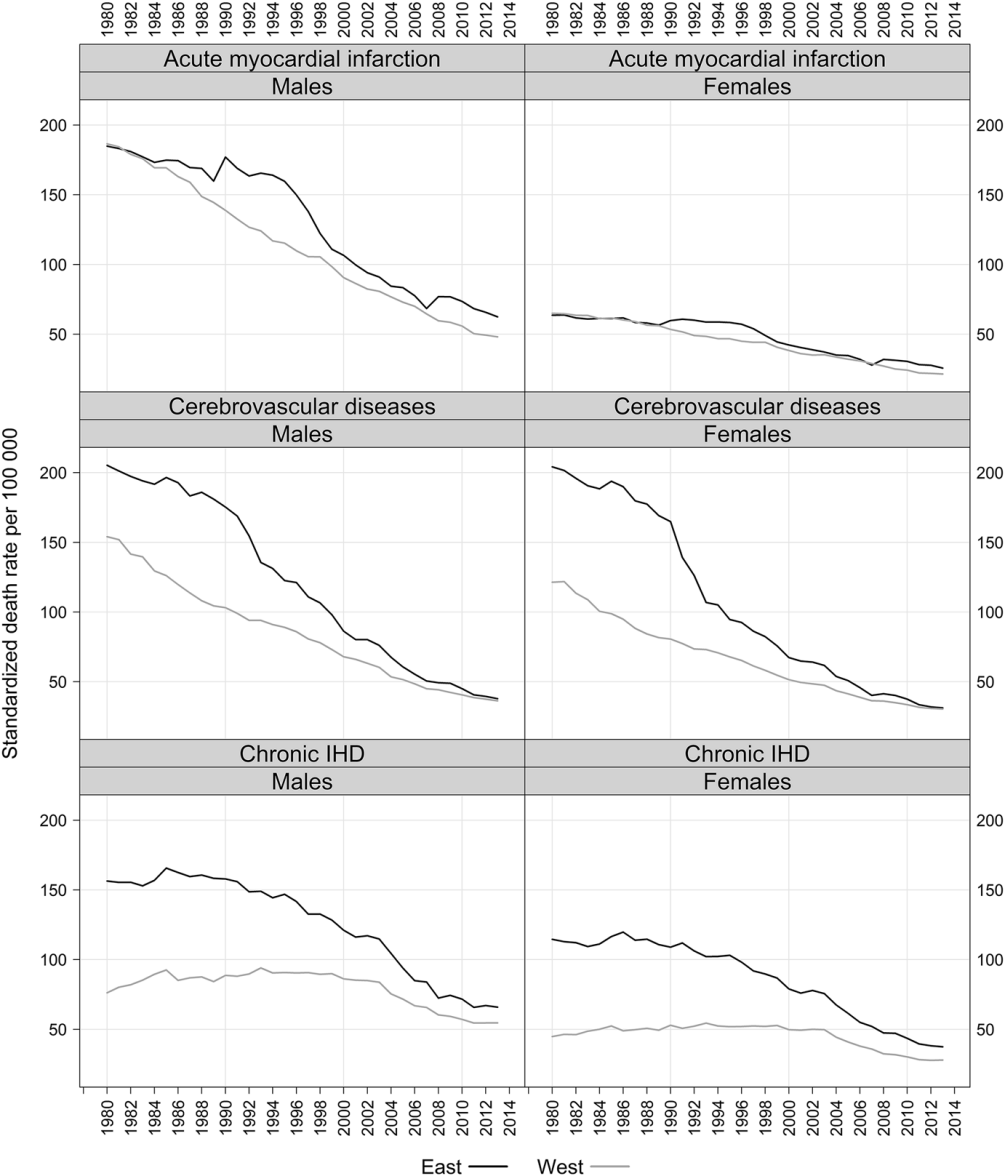
SDR from selected cardiovascular causes, East and West Germany, 1980–2013. *Source*: Reconstructed mortality series by causes of death

Changes in cardiovascular mortality have been the main drivers in the convergence process, particularly among females. This pattern has been previously demonstrated by other studies (Nolte et al. 2000b; Luy 2004) and was confirmed by the analysis presented above. But what does the evolution of more specific causes within the chapter of cardiovascular diseases look like? Figure 8 shows mortality trends from selected cardiovascular conditions: acute myocardial infarction, cerebrovascular diseases, and chronic ischaemic heart diseases.

In 1980, there was no East–West difference in mortality from acute myocardial infarction. However, the gap in mortality from this cause started growing in the middle of the 1980s among males (and in 1990 among females) because of both the rapid improvements in West Germany and the interruption in the declining trend in East Germany. In the East, the rate of mortality increase was particularly high among young males around the time of reunification. For example, between 1989 and 1990 mortality from acute myocardial infarction increased 48% among men aged 35–39, but less than 10% among men aged 70 and older. By the end of the 1990s, mortality levels from this cause of death had converged in East and West Germany.

Unlike for mortality from acute myocardial infarction, East Germans (and particularly females) had a notable disadvantage in mortality from cerebrovascular diseases starting in the 1980s. Moreover, unlike trends in mortality from many other causes, trends in mortality from cerebrovascular diseases showed no signs of deteriorating or even stagnating around the time of reunification. On the contrary: the steady improvements in mortality from this cause observed in the 1980s accelerated in the 1990s. The decline in female mortality was especially impressive: as early as in 1992, the level of female mortality from cerebrovascular diseases had fallen by more than 50%. By the mid-2000s, the trends in cerebrovascular mortality had completely converged in East and West Germany. Currently, there are no differences in mortality levels from this cause either between East and West or between men and women.

The analysis of mortality trends by age (not shown here) suggests that the continuous improvement in mortality from cerebrovascular diseases was driven by the reduction in mortality rates at very advanced age groups. For example, the rapid decline in female mortality in the early 1990s noted above was determined by women aged 85 and older. Interestingly, the mortality declines in the adjacent age groups of 70–74, 75–79, and 80–84 did not exhibit such a rapid decline.

The East–West convergence in mortality from chronic ischaemic heart diseases was slower, mainly because of the large initial disadvantage among East Germans. In the East in the 1980s, mortality from this cause of death was twice as high among males and three times as high among females as in the West. Despite the continuous decline in East Germany since the mid-1980s and the stagnation in West Germany, there was still a large East–West gap in mortality from this cause in the early 2000s. Since then, however, progress has been observed in both parts of Germany, and it is likely that the trends will converge in the near future.

#### Causes of Death Amenable to Health Care

**Fig. 9:**
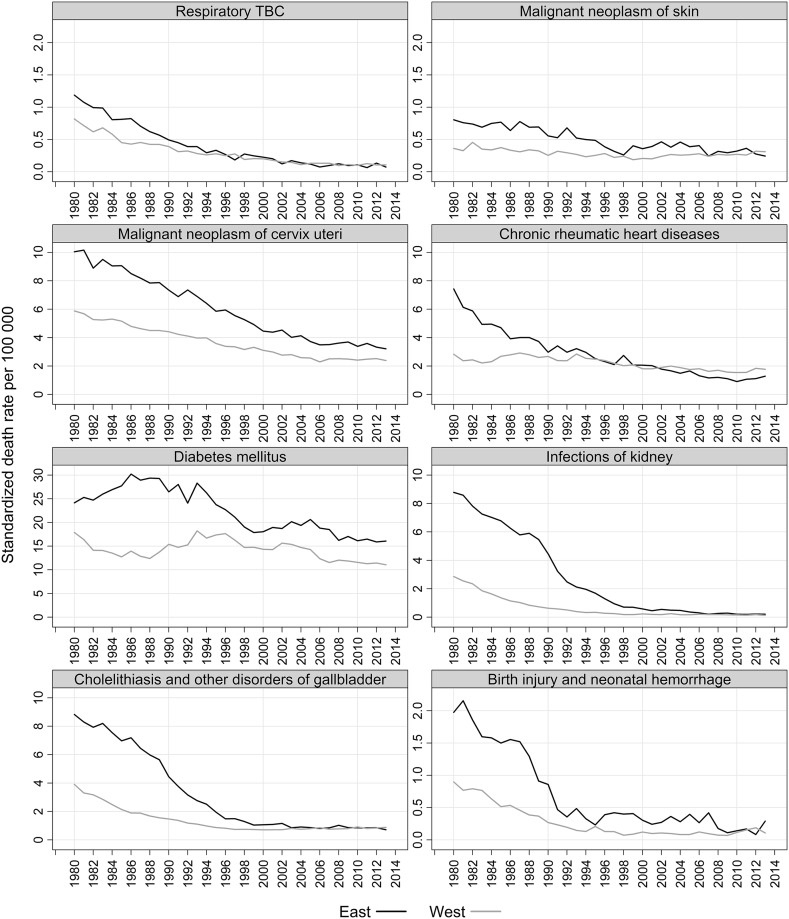
SDR from selected causes amenable to health interventions, East and West Germany, 1980–2013, females. *Source*: Reconstructed mortality series by causes of death

We continue with analysing mortality trends for some diseases amenable to appropriate prevention and medical treatment (Fig. 9). Because deaths from such causes are generally avoidable (until certain ages), it is often assumed that they would not occur if people had adequate health care (Nolte and McKee 2004). Although these causes of death have negligible effects on overall mortality trends and levels, they appear to reflect changes in the quality and efficiency of health care provision. The large differences we see in the initial mortality levels clearly show that medical care was more advanced in the FRG than in the GDR. At the same time, the trends in these causes of death do not indicate that there was a serious crisis in health care provision in the GDR. On the contrary: mortality from all of the selected conditions except diabetes had been declining since the beginning of our observation period, with an obvious acceleration occurring after reunification.

## Discussion

The analysis of long-term mortality trends by causes of death has long represented an important and missing piece of the East–West mortality convergence ‘puzzle’. Such an analysis allows us to disentangle the processes that started in the past from those that emerged after the reunification of Germany. The main advantage of the analysis presented in this paper is that it was based on harmonised cause-specific mortality data that were adjusted for the incorrect coding of important pathologies in the GDR before reunification. In addition, compared to other studies, our analysis took a much broader perspective on the topic, as it employed much longer mortality series and more specific causes of death. Furthermore, in addition to documenting important discontinuities in cause-specific mortality series, we have proposed reasonable solutions for the correction of these discontinuities. Finally, an important advantage of this study is that it was based on adjusted data on population exposures that have just been released (HMD 2016). It has, however, been shown that using the new population exposures for computing mortality rates does not result in significant changes in life expectancy (Kluesener et al. unpublished manuscript). The results of our own analysis confirm that the adjustments for the incorrect estimation of migration flows in the 1990s have a negligible impact on mortality indicators. Thus, the suggestion that these estimates represent a ‘statistical artefact’ can be confidently excluded from the potential explanations for mortality improvements in East Germany.

The discussion is structured into two main parts: first we discuss the principal findings and their relevance to the main hypotheses on the causes of the convergence in health and mortality in East and West Germany. Second, we address the unresolved methodological issues related to the quality of cause-specific mortality data in East Germany.

### Factors Behind East–West Mortality Divergence and Convergence

#### Health Care

It has been suggested that the East–West mortality gap in the 1980s was largely attributable to differences in health care provision and that the rapid adoption of the modern health care system of West Germany starting in 1990 resulted in immediate health improvements in East Germany (Nolte et al. 2002). The GDR health care system was much less efficient than the West German system in tackling non-communicable diseases. Moreover, the East German system suffered not only just from a lack of sophisticated medical equipment and modern hospitals, but also from shortages of essential medical supplies (Mielck 1991). Meanwhile, in West Germany, several successful reforms to improve access to medical care, while keeping medical costs at publicly acceptable levels, were implemented in the 1970s and the 1980s (Iglehart 1991). From the mid-1980s to the mid-1990s, both primary and secondary preventive coronary care improved substantially (Müller-Nordhorn et al. 2004). For example, the number of cardiac catheterisation procedures performed increased from 45,000 in 1984 to 450,000 in 1996, and the number of angioplasties performed increased by about 50-fold (Perleth et al. 1999). These improvements clearly contributed to the rapid decline in cardiovascular mortality observed in West Germany and, consequently, to the widening gap between the GDR and the FRG, which approached its peak in the late 1980s.

We have shown that among males, roughly half of this gap was attributable to higher mortality from heart diseases and to the age groups 60 and older in East Germany. Among females, the mortality gap was almost exclusively driven by East–West differences in mortality from cerebrovascular and heart diseases and to the age groups 60 and older. Although these observations point to shortcomings in the GDR health care system, it is also worth noting that the mortality trends in East Germany were not worsening. On the contrary: in the 1980s, life expectancy at birth was growing among both males and females. Our analysis has shown that this improvement was largely due to the reduction in cerebrovascular mortality among the elderly. Given the evidence of the deterioration in the quality of health care provision in the GDR, this observation is hard to explain. One possible explanation is that, despite its many deficiencies, the GDR health care system was still able to deliver services at a level that was sufficient to force slow mortality improvements. It has been suggested that even in the 1970s, when fewer monetary resources were allocated to health, the GDR system provided health services of good quality because the benefits of the limited care provided were distributed relatively equally among all population groups (Roemer 1985). We have shown that the causes of death amenable to medical care had very positive dynamics even before reunification. In addition, the results of the WHO MONICA Project, which was conducted in the GDR in 1988–1990 and in the FRG in 1985–1987, show that the success of acute coronary care (as indicated by the weighted coronary treatment score) in the East and West German MONICA centres was very similar (Tunstall-Pedoe et al. 2000).

#### Lifestyles and Risk Factors

Lifestyle changes have been shown to be another important group of factors responsible for the sustained health improvements in East Germany (Diehl 2008; Kibele 2012). Heinemann et al. (1995) argued that high-technology medicine in West Germany, such as sophisticated diagnostics and surgical treatment, reduced case fatality to a limited extent only and that the causes of the East–West mortality differential are likely to be more associated with lifestyle-dependent risk factors. Evidence from the WHO MONICA Project suggests that the GDR was in a relatively unfavourable position in terms of well-known coronary risk factors. Shortly before reunification, high prevalence rates of hypercholesterolemia, hypertension, diabetes, and obesity were reported among both males and females in East Germany (Heinemann et al. 1998; Thamm 1999). However, any comparison of the risk profiles of East and West Germany should be made with caution, given the differences in the study designs, the analytical methods, and the measurements applied (Jaross et al. 1994). Based on their review of small-scale epidemiological studies conducted in the GDR between 1968 and 1992, Heinemann and colleagues (1995) concluded that the major cardiovascular risk factors of East Germans were deteriorating. On the other hand, the results of a comparative study of risk factors for coronary heart disease conducted in Dresden (GDR) and Muenster (FRG) did not sufficiently explain the differences in morbidity and mortality in East and West Germany (Jaross et al. 1994).

Nevertheless, it is likely that there were differences in the risk profiles of the populations of the two German states and that these divergent risk profiles contributed to the East–West mortality gap. However, these differences do not explain the widening gap in the late 1980s. We have shown that particularly in the case of males, the accumulated disadvantage among East Germans was largely driven by mortality from external causes, which was falling rapidly in the West and was stagnating in the East.

The role of mortality from external causes of death became even more important after reunification but again, mainly among males. In this case as well, the relative disadvantages among Easterners were primarily due to notable improvements among Westerners. The sudden jump in mortality observed in East Germany in 1990 is an exception. Sharp increases in mortality from alcohol-related causes, such as liver cirrhosis and alcohol abuse, point to the role of alcohol consumption. It is also likely that alcohol was involved in the increasing rates of mortality from traffic accidents. There is limited evidence suggesting that the high level of per capita alcohol consumption in East Germany around the time of reunification (15.5 litres in 1989) subsequently declined (to 12 litres in 1993 and to nine litres in 1995) (Riphahn 1999). Alcohol consumption appears to have been linked to psychosocial stress related to concerns about the consequences of the transition from a socialist to a market economy, such as anxiety about becoming unemployed or having an uncertain future (Wiesner 1992). As we have shown, however, the rise in psychosocial stress was not reflected in suicide mortality trends, which have been improving steadily in East Germany since the 1980s.

The rapid change in dietary habits appears to have been a very important factor in the improvement in the cardiovascular profile of East Germans after reunification and thus in mortality reductions. The GDR population consumed more butter and sausage, and far fewer vegetables and tropical fruits than the FRG population. As the availability of vegetables and fruits improved after 1989, East Germans started adopting a healthier diet (Winkler, et al. 1997). Similar positive developments accompanied by notable reductions in cardiovascular mortality have been observed in other post-communist countries, such as Poland and the Czech Republic (Zatoñski and Boyle 1996; Bobak et al. 1997). Thus, the changes in the dietary habits of East Germans appear to be more attributable to economic liberalisation than to reunification.

Differences in past smoking habits may have also contributed to changes in the East–West mortality gap (Myrskylä and Scholz 2013). While the pre-unification data suggest that there was almost no difference in the smoking prevalence of East (42%) and West German (41%) males, East German females (19%) had a more favourable profile than West German women (27%). These differences were driven by people under age 45, as the East–West differences among people aged 45 and older were small (Heinemann et al. 1996). Thus, younger East German female cohorts might have been the only contributors to the rapidly narrowing gap in the 1990s. At that time, however, they were still ‘too young’ to play a decisive role in the convergence process.

#### Concluding Remarks

The mortality trends in East and West Germany provide an illustrative example of the divergence–convergence cycles in mortality (Vallin and Meslé 2004). The ‘pioneering’ role played by West Germany resulted in a phase in the 1980s during which the two states diverged, followed by a phase in the 1990s during which East Germany was ‘catching up’ with the West. However, while East–West mortality among women has fully converged, a gap mainly driven by mortality at working ages can still be observed among males. While the reasons for this gap, which has lasted more than 15 years, are not fully understood, there is some evidence that economic factors have been especially important contributors to its persistence (Scholz 2010).

The cause-specific analysis revealed that there were three distinct historical processes taking place around the time of reunification in East Germany: (1) a continuous mortality reduction before reunification, (2) a temporary increase in mortality related to the abrupt social transition (which mostly affected males of working ages and socially sensitive causes, such as accidents, alcohol-related diseases, and acute myocardial infarction) in 1990–1991, and (3) a reunification-driven process of convergence that was mostly caused by the accelerated decline in mortality from cerebrovascular and chronic heart diseases.

Rapid progress in the prevention and the treatment of cerebrovascular diseases, as well as the continuous reduction in mortality from external causes in West Germany in the 1970s and the 1980s, largely explains the accumulated advantages of West Germans. Prior to reunification, the situation in East Germany was not deteriorating and was even improving at a slow pace. Mortality improvements in the GDR, which started in the 1980s, might be interpreted as the first signs of a cardiovascular revolution. The process of a sustained reduction in cardiovascular mortality is driven by both fundamental changes in behavioural risk factors and advancements in medical technology and disease prevention (Vallin and Meslé 2004; Grigoriev et al. 2014). In the case of East Germany, shifts in individual behaviour likely started before reunification, while the real progress in medical care occurred later with the implementation of the Western system of health care.

Thus, German reunification per se did not initiate the convergence process, but rather reinforced and accelerated trends that were already apparent. The experiences of other post-communist countries—such as the Czech Republic, Poland, and Slovenia—show that mortality improvements could be achieved without the massive investments in health care that were made in East Germany. It is true that the progress observed in East Germany was faster than in these other countries. However, it is also true that before the collapse of the communist system, the GDR had a better starting position, with more favourable mortality levels. It therefore appears that East Germany was more predisposed to making rapid progress than other Eastern Bloc countries at the time of the fall of the Berlin Wall.

### Methodological Issues and Data Quality

In this paper, we reported the initial results of our ongoing work on the reconstruction of continuous mortality trends by causes of death in East and West Germany. Currently, our work is mainly focused on the complex issue of the transition to the adoption of FRG coding practices, which occurred in East Germany in October 1990. This transition brought about unprecedented changes in the system of cause-of-death data collection in East Germany and introduced severe discontinuities in mortality trends. Unfortunately, the breaks could not be treated by applying the ‘classical’ method of a posteriori reconstruction (Meslé and Vallin 1996). To address this issue, we applied correction factors derived from a thorough examination of time trends. The amount of our correction for cancer mortality, of about 10%, is consistent with the results of an autopsy study conducted in the Goerlitz municipality in 1987, which found roughly the same amount of underestimation of cancers (Modelmog et al. 1992). The corrections within the chapter on cardiovascular diseases are also in line with the results of the study on the validity of cardiovascular diagnoses in the GDR, which suggest that there were significant (roughly twofold) upward corrections of deaths from myocardial infarction and cerebrovascular diseases (Heinemann et al. 1998).

Despite these adjustments, the mortality data for East Germany are still not problem-free. The reliability of mortality data from the principal cause of death, cardiovascular diseases, is of major concern. We applied the corrections within the chapter, but we were unable to come up with a justifiable solution for the downward correction of the chapter as a whole. Meanwhile, there is evidence of the overestimation of CVD mortality in the GDR (Modelmog et al. 1992), which our own observations have confirmed. For example, it is very likely that the abrupt decline in female mortality from cerebrovascular diseases in the early 1990s driven by the age group 85 and older was at least partially an artefact caused by the adoption of Western coding practices.

Like the trends in CVD mortality, the trends in mortality from digestive diseases have to be interpreted with caution. As we have shown, during the 1980s mortality levels from this cause of death were suspiciously low relative to the levels observed in the 1960–1970s and the 1990s. Our finding that in the 1980s liver cirrhosis and other conditions related to alcohol were included in the list of ‘hidden’ (‘politically undesirable’) causes of death might explain why mortality from digestive diseases was underestimated. Moreover, the increase in mortality from chronic liver disease and cirrhosis in 1990–1991 was too abrupt to be fully attributable to changes in alcohol consumption. Our hypothesis is consistent with the results of the Goerlitz study, which showed that mortality from digestive diseases was underestimated by about 20% (Modelmog et al. 1992).

### Electronic Supplementary Material

Below is the link to the electronic supplementary material.

Supplementary material 1 (DOCX 1443 kb)

## Appendix 1

See Table 2.

**Table 2:** List of causes of death used in the analysis with the correspondences to the ICD-10

	M List	ICD-10
Infectious and respiratory diseases	1–25, 101–115	A00-B99, J00-J99
Respiratory tuberculosis (TBC)	7	A15-A16, J65
Neoplasms	26–55	C00-D48
Malignant neoplasm of the skin	36	C44
Malignant neoplasm of the cervix uteri	39	C53
Diseases of cardiovascular system (CVD)	77–100	I00-I99, G45
Heart diseases	77–92	I00-I02, I05-I13, I15, I20-I22, I24-I28, I30-I31, I33-I38, I40, I42, I44-I51
Chronic rheumatic heart diseases	78	I05-I09
Acute myocardial infarction	84	I21-I22
Chronic ischaemic heart diseases (IHD)	86	I20, I25
Cerebrovascular diseases	93–95	I60-I69, G45
Other CVD diseases	96–100	I70-I78, I80-I99
Diseases of the digestive system	116–124	K00-K99
Chronic liver diseases and cirrhosis	122	K70, K73, K74
Cholelithiasis and other disorders of the gallbladder	123	K80-K82
External causes of death	156–186	V00-Y99
Motor accidents	156	V02-V04, V09, V12-V14, V20-V79
Suicide	168-172	X60-X84
Remaining causes of death	REST	REST
Diabetes mellitus	57	E10-E14
Alcohol abuse	65	F10
Infections of kidney	132	N10-N12, N15
Birth injury and neonatal haemorrhage	141	P03, P10-P15, P50-P52, P54

## Appendix 2

See Table 3.

**Table 3:** Age–cause contributions to the East–West gap in life expectancy at birth by periods. *Source*: Own calculations based on the reconstructed data

Period	Age group	Causes of death								
		INFRES	NEO	HD	CRVD	OTHCVD	DIG	EXT	REST	TOT
A. Males										
1980	0–14	− 0.01	0.00	0.01	0.00	0.00	0.01	− 0.02	0.04	0.03
	15–29	0.03	0.02	0.02	0.00	0.00	0.00	− 0.11	0.03	− 0.01
	30–44	0.00	0.03	0.01	− 0.01	0.00	− 0.02	0.09	0.00	0.10
	45–59	0.07	0.05	0.05	0.00	0.01	− 0.04	0.06	0.02	0.22
	60–74	0.12	− 0.03	0.25	0.01	0.04	− 0.02	0.04	0.06	0.47
	75+	0.03	− 0.11	0.25	0.11	0.06	− 0.01	0.01	0.02	0.36
	Total	0.24	− 0.04	0.59	0.11	0.11	− 0.08	0.07	0.17	1.17
1990	0–14	0.00	0.01	0.03	0.00	0.00	0.01	0.10	0.00	0.15
	15–29	0.00	0.02	0.02	0.01	0.00	0.02	0.27	− 0.02	0.32
	30–44	− 0.03	0.02	0.11	0.01	0.01	0.13	0.25	0.08	0.58
	45–59	0.04	0.08	0.30	0.06	0.01	0.15	0.23	0.12	0.99
	60–74	0.06	0.02	0.51	0.13	0.00	0.06	0.06	0.08	0.92
	75+	− 0.02	− 0.10	0.30	0.22	0.00	0.00	0.03	0.00	0.43
	Total	0.05	0.05	1.27	0.43	0.02	0.37	0.94	0.26	3.39
2000	0–14	0.00	0.00	− 0.01	0.00	0.00	0.00	0.02	− 0.01	0.00
	15–29	0.00	− 0.01	0.01	0.00	0.00	0.01	0.16	− 0.05	0.12
	30–44	− 0.01	0.02	0.04	0.00	0.00	0.13	0.11	0.03	0.32
	45–59	− 0.02	0.04	0.08	0.01	0.01	0.17	0.10	0.05	0.44
	60–74	− 0.04	0.13	0.14	0.04	0.00	0.07	0.02	0.03	0.39
	75+	− 0.06	− 0.01	0.14	0.12	0.01	0.00	0.01	− 0.02	0.19
	Total	− 0.13	0.17	0.40	0.17	0.02	0.38	0.42	0.03	1.46
2013	0–14	0.00	− 0.01	0.00	0.00	0.00	0.00	0.01	− 0.05	− 0.05
	15–29	0.00	0.00	0.00	0.00	0.00	0.00	0.05	− 0.01	0.04
	30–44	0.00	0.03	0.04	0.00	0.00	0.05	0.05	0.01	0.18
	45–59	0.00	0.17	0.14	0.02	0.00	0.15	0.05	0.06	0.59
	60–74	− 0.03	0.10	0.15	0.01	− 0.02	0.06	0.03	0.00	0.30
	75+	− 0.05	0.07	0.24	0.00	− 0.05	− 0.01	0.02	− 0.07	0.15
	Total	− 0.08	0.36	0.57	0.03	− 0.07	0.25	0.21	− 0.06	1.21
*B. Females*										
1980	0–14	0.01	0.00	0.00	0.00	0.00	0.02	− 0.07	− 0.03	− 0.07
	15–29	0.00	0.00	0.00	0.00	0.00	0.00	0.00	0.03	0.03
	30–44	0.00	0.04	0.02	− 0.02	0.00	− 0.04	0.05	0.01	0.06
	45–59	0.02	0.06	0.10	0.01	0.01	− 0.02	0.04	0.04	0.26
	60–74	0.04	− 0.02	0.43	0.17	0.07	0.04	0.04	0.11	0.88
	75+	0.02	− 0.12	0.38	0.52	0.13	0.00	0.00	0.01	0.94
	Total	0.09	− 0.04	0.93	0.68	0.21	0.00	0.06	0.17	2.10
1990	0–14	0.02	0.01	0.02	0.00	0.00	0.01	0.05	− 0.01	0.10
	15–29	− 0.01	0.02	0.00	0.00	0.00	0.01	0.08	0.01	0.11
	30–44	0.00	0.00	0.03	0.01	0.00	0.02	0.06	0.02	0.14
	45–59	0.01	0.03	0.14	0.04	0.00	0.05	0.08	0.07	0.42
	60–74	0.02	0.00	0.46	0.29	0.02	0.06	0.06	0.11	1.02
	75+	− 0.04	− 0.08	0.36	0.68	0.01	0.00	0.07	− 0.02	0.98
	Total	0.00	− 0.02	1.01	1.02	0.03	0.15	0.40	0.18	2.77
2000	0–14	0.01	0.00	0.00	0.00	0.00	0.00	0.01	− 0.07	− 0.05
	15–29	0.00	0.00	0.00	0.00	0.00	0.00	0.04	0.00	0.04
	30–44	− 0.02	− 0.02	0.00	0.00	0.00	0.06	0.01	0.00	0.03
	45–59	− 0.05	− 0.12	0.04	0.00	0.00	0.09	0.04	− 0.02	− 0.02
	60–74	− 0.05	0.02	0.13	0.06	0.00	0.04	0.02	0.04	0.26
	75+	− 0.10	− 0.04	0.17	0.22	0.02	− 0.02	0.02	− 0.03	0.24
	Total	− 0.21	− 0.16	0.34	0.28	0.02	0.17	0.14	− 0.08	0.50
2013	0–14	0.01	0.00	0.00	0.00	0.00	0.00	0.00	− 0.05	− 0.04
	15–29	− 0.07	− 0.02	− 0.02	− 0.01	0.02	0.07	0.18	− 0.14	0.01
	30–44	0.00	0.01	0.00	0.00	0.00	0.01	0.01	0.00	0.03
	45–59	− 0.02	− 0.02	0.03	0.00	0.00	0.04	0.01	0.01	0.05
	60–74	− 0.05	− 0.08	0.05	0.00	− 0.01	0.01	0.01	− 0.01	− 0.08
	75 +	− 0.11	− 0.03	0.35	0.03	− 0.07	− 0.03	0.04	− 0.14	0.04
	Total	− 0.24	− 0.14	0.41	0.02	− 0.06	0.10	0.25	− 0.33	0.01

*INFRES* infectious and respiratory diseases, *NEO* neoplasms, *HD* heart diseases, *CRVD* cerebrovascular diseases, *OTHCVD* other CVD diseases, *DIG* digestive diseases, *EXT* external causes of death, *REST* other (residual) causes, *TOT* all causes combined
