# Supplementary material for: Health Convergence Between East and West Germany as Reflected in Long-Term Cause-Specific Mortality Trends: To What Extent was it Due to Reunification?
Source: Eur J Popul. 2017 Dec 4;33(5):701–31. doi: 10.1007/s10680-017-9455-z (PMC5740210; doi:10.1007/s10680-017-9455-z)
Supplement: Supplementary file 1 — Supplementary material 1 (DOCX 1443 kb) [file 10680_2017_9455_MOESM1_ESM.docx]

**Online supplementary material**

**Corrections for the major distortions caused by the introduction of new coding practices in East Germany in 1990**

Based on a thorough inspection of the trends for each cause of death and a comparison of these trends with trends in West Germany, we identified three types of effects of the 1990 coding change. We can see that immediately after the new coding system had been introduced, there was a peak in the major ill-defined categories: i.e., heart failure, pneumonia, and unknown cause. This peak suggests that the coding transition was accompanied by initial uncertainty about the selection of the underlying cause of death. The second type of effect was limited to circulatory conditions, and suggests that there was a complete change in the medical approach to the coding of different categories of circulatory diseases. Finally, the third type of effect was related to cancer. This shift was the most complex, because it affected a large number of non-cancer causes of death across the entire classification.

The 1990 coding change seriously affected a large number of circulatory disease categories. The main items that were used less frequently after 1990 were atherosclerosis and essential hypertension. Although both of these causes are, under the WHO coding rules, considered undesirable choices when reporting the underlying cause of death, they had long been over-reported in the Eastern European countries, including in the republics of the USSR. Our correction to circulatory categories was therefore based on a careful reattribution of deaths from atherosclerosis and essential hypertension in 1980-1989 and deaths from heart failure in 1990 to better defined circulatory conditions: i.e., to acute myocardial infarction, chronic ischemic heart disease, haemorrhagic stroke, and other cerebrovascular diseases. For each target cause, we assessed the size of the jump observed between 1991 and 1992, and we estimated a proportion (by age and sex) to be taken from either of the two ill-defined source categories (atherosclerosis and/or essential hypertension). Figures S1 and S2 display the results of these individual corrections for males and females, respectively.


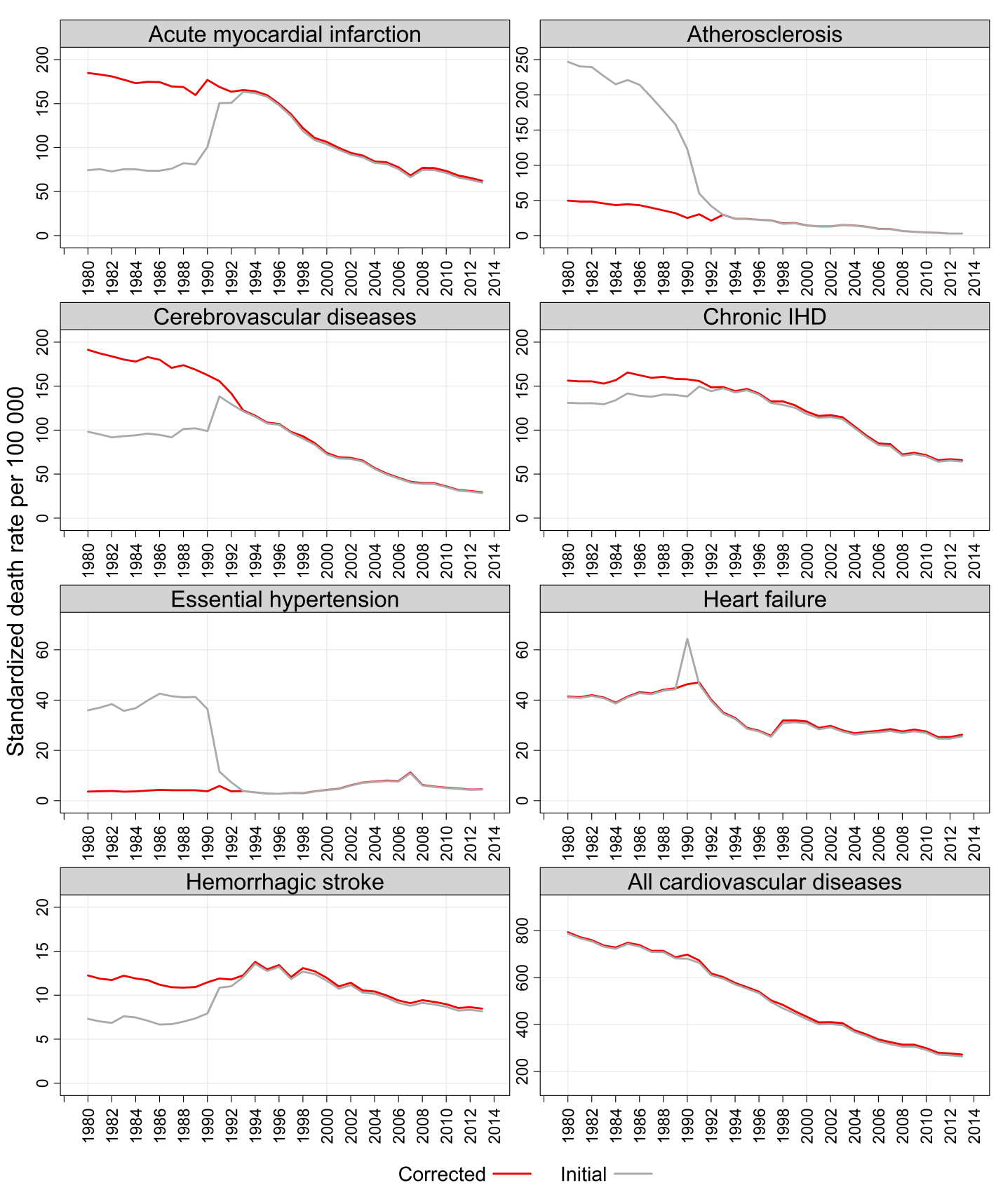


**Figure S1. Corrections of mortality from cardiovascular diseases; East Germany, males**

**
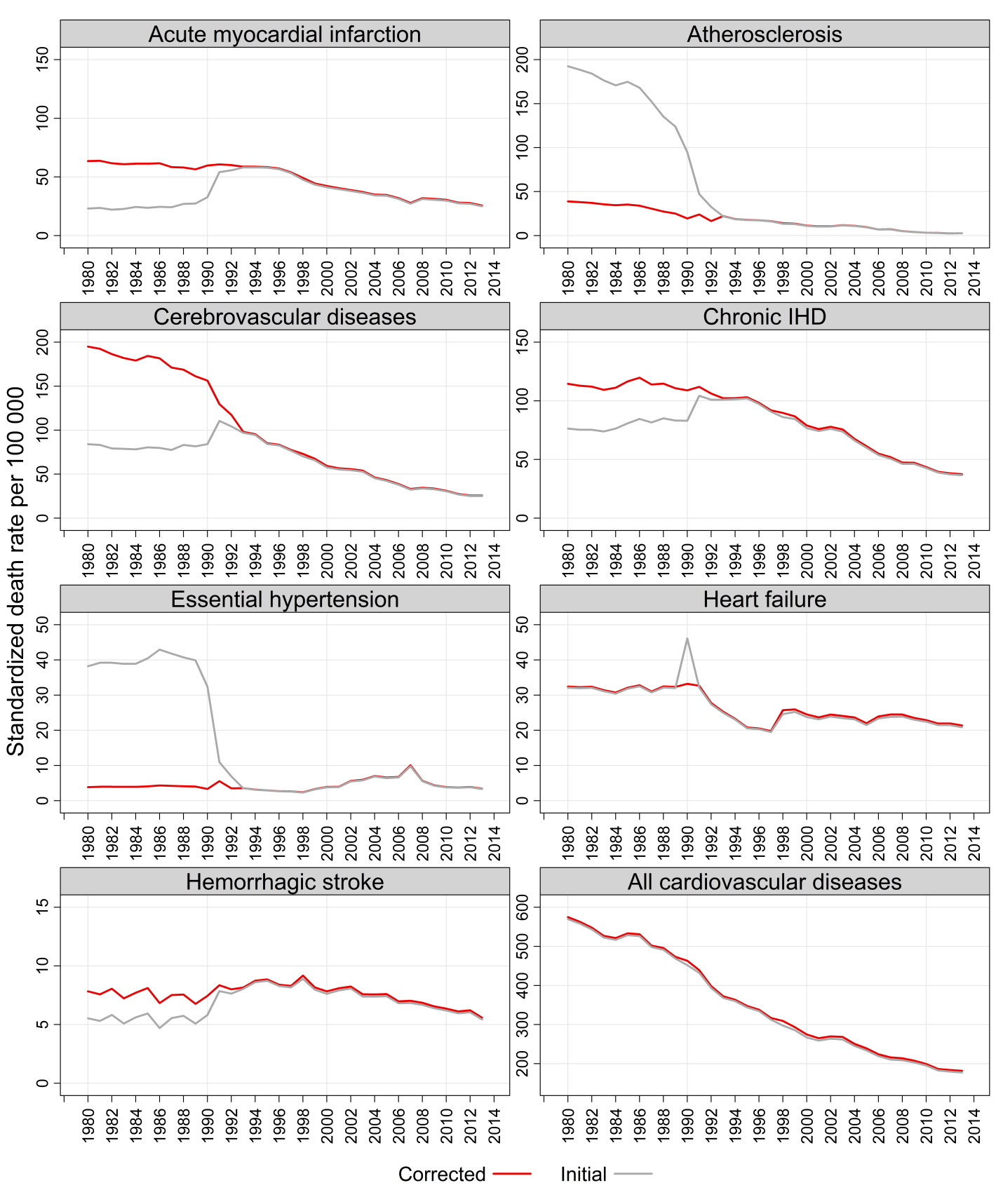
**

**Figure S2. Corrections of mortality from cardiovascular diseases; East Germany, females**

*Under-reporting of cancer*

As an examination of cancer trends indicates that they were seriously disrupted at the level of the whole chapter, it is clear that corrections are needed across the whole classification. The last panels of Figures S3 (males) and S4 (females) show that the impact of the new coding system on cancer was stepwise: a single-year drop in 1990 was followed by an increase starting in 1991.

**
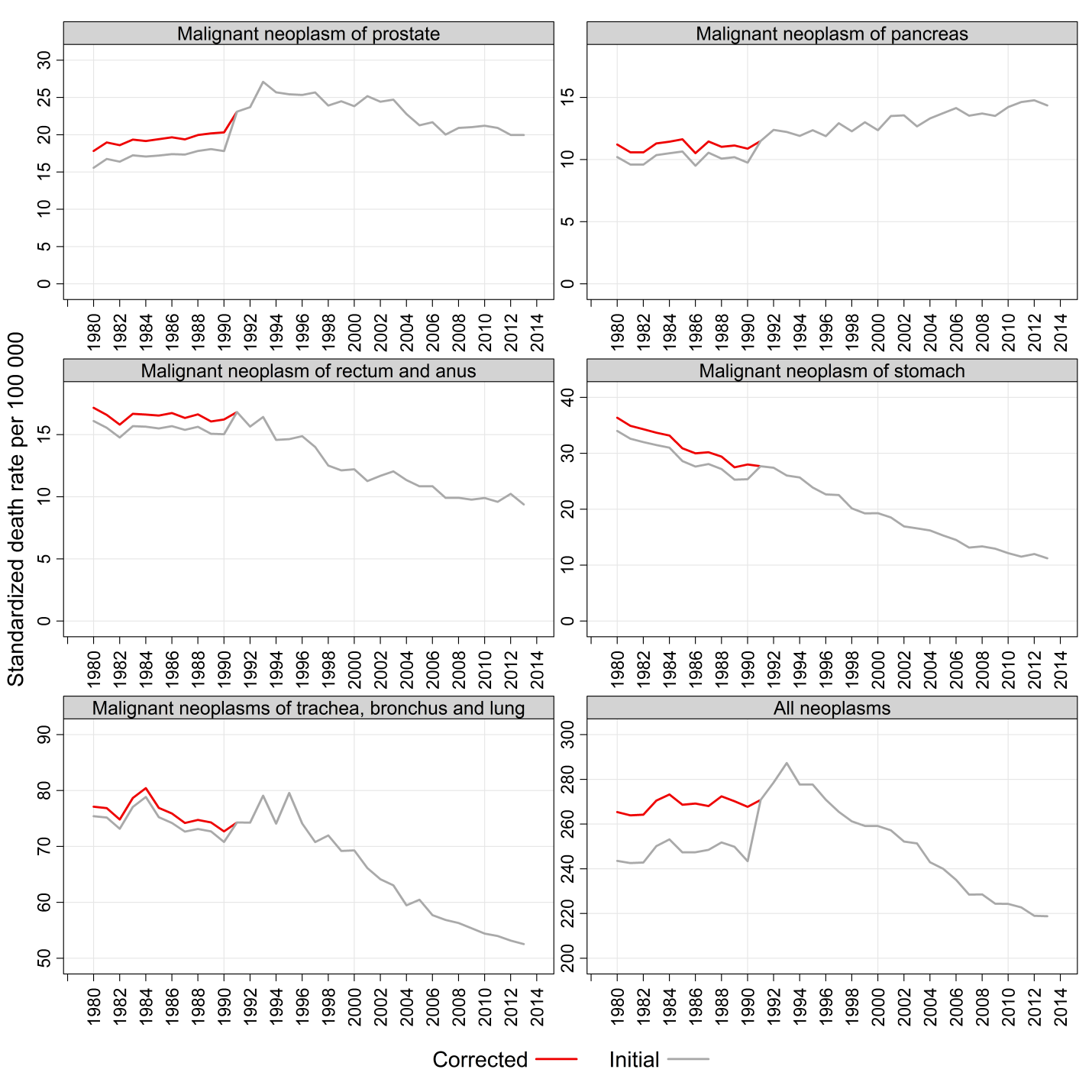
**

**Figure S3. Corrections of selected cancers; East Germany, males**

A sudden decrease in neoplasms in 1990 clearly corresponds to the peak observed in ill-defined and unknown causes; this development suggests that there was uncertainty surrounding the expected change in cancer coding in the upcoming year. To place the increase in 1991 into context, we performed a thorough inspection of all cause-specific trends. This allowed us to identify several conditions with trends in 1991 that were symmetrical to the clear and sustained decrease in the trend in neoplasms. These conditions include: malnutrition, other endocrinologic and metabolic diseases, anaemias, other mental disorders, diseases of the eye and adnexa, diseases of the skin, other musculoskeletal diseases, complications of medical care, and symptoms and signs. The shift away from reporting these conditions as the cause of death and towards reporting cancer was probably related to the abovementioned obligation of the certifying practitioner to fill out an extra form for cancer deaths. It appears that physicians in the GDR adopted different strategies to avoid having to code for cancer: they either selected an ill-defined category (symptoms and signs); or they selected a condition that was present at the moment of death, but that was not the underlying cause based on the WHO rules for identifying the main cause of death (e.g., end-stage conditions like malnutrition or decubitus, or chemotherapy-related conditions like anaemia). This assumption also finds support in recent studies on multiple causes of death, which show significant associations between cancer and the conditions listed above (Pechholdová, 2014). Moreover, a comparison with corresponding data from West Germany yielded proof of the over-reporting of all of these causes between 1980 and 1990.

Even though there are clear indications of correspondences with cancer at the statistical level, there is no information at the level of medical content about the precise cancer types to which these diseases should be reattributed. In our present approach, we estimated the level of over-reporting for each of these conditions, subtracted and summarised the excess deaths in 1980-1990, and redistributed them across individual cancer sites based on the relative size of the disruption. Figures S3 and S4 show the trends in the most affected cancer types before and after the correction, as well as across the whole chapter. So far, however, our corrections have been made at the expense of causes for which the trends before 1990 were clearly implausible. Making further corrections would require us to take a more speculative approach.

**
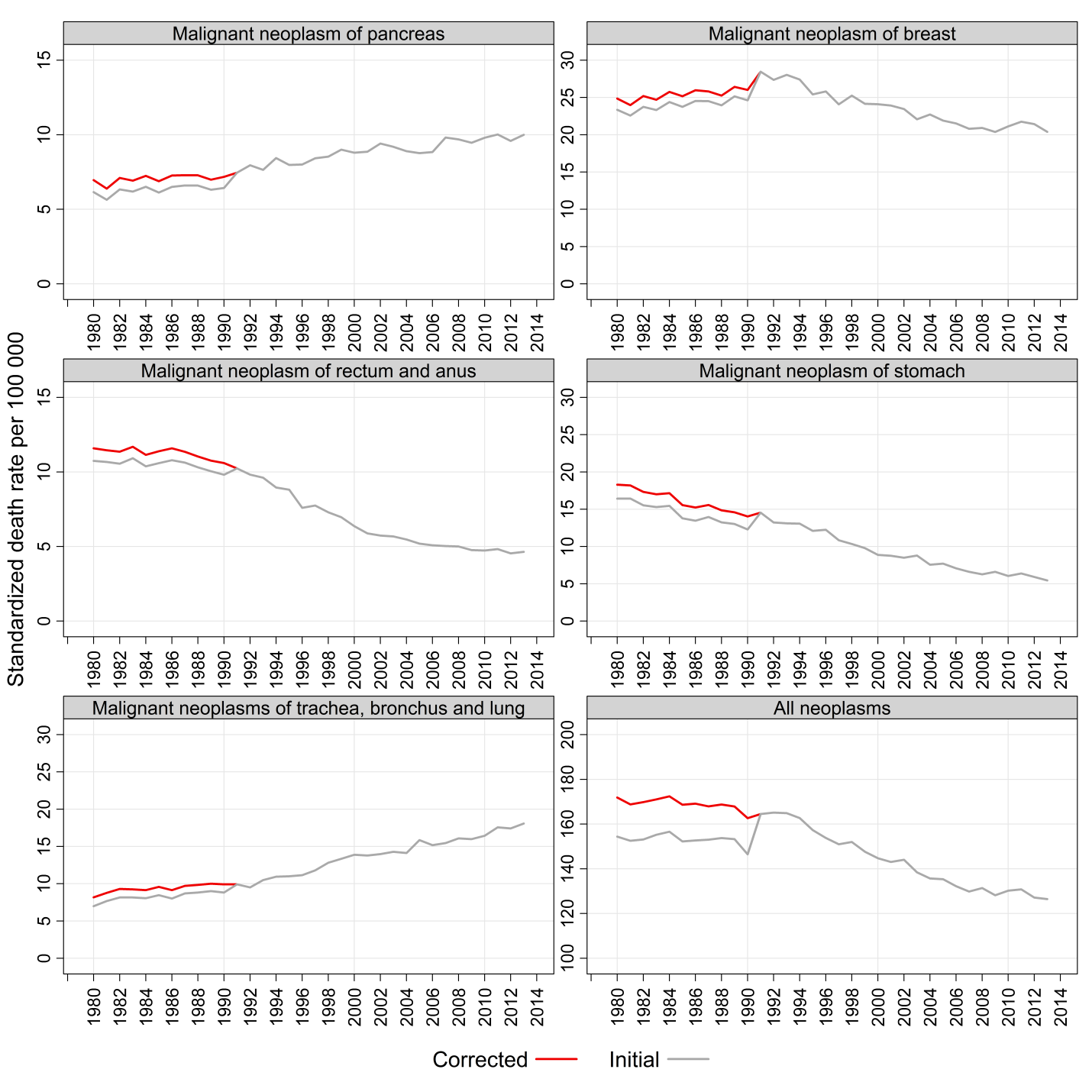
**

**Figure S4. Corrections of selected cancers; East Germany, females**
